# Management of Intra-operative Contamination of an Anterior Cruciate Ligament Reconstruction Graft: A Cross-Sectional Survey of UK Orthopaedic Surgeons

**DOI:** 10.7759/cureus.108309

**Published:** 2026-05-05

**Authors:** Hassan Kazemi, Josh Henry, Jack A Turnbull, Neil Jain

**Affiliations:** 1 Trauma and Orthopaedics, Royal Bolton Hospital, Bolton, GBR; 2 Orthopaedics, Royal Oldham Hospital, Oldham, GBR; 3 Trauma and Orthopaedics, Health Education England North West, Manchester, GBR; 4 Trauma and Orthopaedics, Pennine Acute Hospitals NHS Trust, Oldham, GBR

**Keywords:** acl, graft contamination, graft decontamination, graft sterilisation, intraoperative contamination

## Abstract

Purpose

Intra-operative anterior cruciate ligament (ACL) graft contamination is a rare but recognised complication. There are no guidelines or consensus on the management of this occurrence. Our aim was to survey trauma and orthopaedic surgeons with a knee subspecialty interest in the United Kingdom (UK) who perform ACL reconstruction (ACLR) to explore the preferred strategy when there is intra-operative graft contamination.

Methods

An online questionnaire was sent to UK ACLR surgeons. The survey included the year of training completion, the average number of ACLs per year, whether they have experienced any intra-operative graft contamination, the strategies implemented, and whether they are aware of any literature and how it has influenced their strategies.

Results

Twenty-eight responses were received, with an average of 11 years of experience in ACLR, totalling an estimated of more than 15,000 ACLR. Three surgeons surveyed had experienced a single intra-operative ACL graft contamination, all of which were soaked in chlorhexidine gluconate (CG) solution. Of those surgeons who had not encountered a contaminated graft, 11 (44%) would use CG, 7 (28%) would soak in saline and vancomycin solution, and 7 (28%) would harvest a new graft.

Conclusions

There is significant variation in management strategy for an intra-operative contaminated graft in ACLR. This variation highlights the need for further work to develop a consensus for guidance for ACLR surgeons.

## Introduction

The consequences of an infected anterior cruciate ligament (ACL) reconstruction (ACLR) include multiple surgical procedures, prolonged antibiotic treatment, inferior functional outcomes, and compromised return to sport [1,2].

Although uncommon, accidental intra-operative graft contamination, most frequently through contact with non-sterile surfaces, such as the operating theatre floor, is a recognised complication and carries a risk of deep joint infection.

The optimal management of a contaminated ACL graft remains unclear. A survey of sports medicine specialists published in 2005 demonstrated marked variability in surgeons' management strategies, reflecting a lack of consensus at that time [3]. An Italian survey of experts in 2020 reflected on how inadvertent graft contamination is a rare, almost once-in-a-career occasion [4], and they also acknowledged the difficulty in planning for a rare, unexpected event.

A growing body of experimental evidence, including a systematic review and meta-analysis [5], has evaluated graft decontamination strategies, with several studies demonstrating that agents such as chlorhexidine gluconate and povidone-iodine are effective in reducing bacterial contamination while preserving the biomechanical properties of tendon grafts [6-11].

Despite this evolving evidence base, no contemporary guidelines exist, and it is unclear whether surgeon practice has changed in response to more recent laboratory and clinical data. The primary objective of the present study was therefore to evaluate current practice among United Kingdom (UK) consultant sports knee surgeons when faced with inadvertent intra-operative ACL graft contamination, and to assess whether their management strategies are influenced by emerging evidence regarding graft decontamination.

By characterising contemporary practice patterns, this study's secondary objectives are to highlight areas of agreement and ongoing variability within the literature and to inform future research and guideline development for this rare but clinically significant intra-operative scenario.

## Materials and methods

A cross-sectional, anonymous questionnaire survey was conducted to explore current management strategies for intra-operative contamination of ACL grafts among consultant sports knee surgeons across the UK.

Surgeons were identified through regional professional networks, conferences, and events. Participation was voluntary, and all responses were collected anonymously. A total of 28 responses were received.

A 10-item questionnaire was developed to assess surgeons' experience and decision-making regarding intra-operative ACL graft contamination. The survey included the following questions: (1) the year of completion of training; (2) the number of years in practise; (3) the average monthly ACLR performed by each surgeon; (4) the average annual ACLR performed by each surgeon; (5) the preferred choice of graft (hamstring, bone-to-bone patella tendon, quadriceps tendon, allograft, synthetic, or other); (6) whether they have experienced intra-operative graft contamination, and how many times; (7) what were the management strategies in such an event, decontamination or another graft; (8) surgeons who did not experience graft contamination before, a hypothetical scenario in the event of graft contamination, and their preferred strategy; (9) whether they have come across any studies describing the impact of graft contamination, including bacterial load, effect on mechanical strength, post-operative infection, and clinical outcomes; and (10) whether the literature has influenced the implemented or hypothetically proposed strategies.

The survey was distributed electronically using an online survey platform, and the link can be found in the supplementary materials. Responses were collected over a defined study period from July 2025 to March 2026 and stored securely. No identifiable personal or patient data were collected.

Data were analysed descriptively. Results are presented as frequencies and percentages. Given the exploratory nature of the study and the sample size, no inferential statistical analyses were performed.

This study surveyed healthcare professionals regarding self-reported clinical practice and did not involve patients, patient data, or identifiable information. Participation was voluntary, and responses were anonymous. In accordance with UK Health Research Authority guidance, formal National Health Service (NHS) Research Ethics Committee approval was not required.

## Results

We received a total of 28 responses to the questionnaire from the orthopaedic knee surgeons in the UK. Training completion ranged from 1998 to 2025, with a mean practice duration of 11 years. The median ACL reconstructions per year among respondents was 40 (interquartile range: 37.5, range: 15-185). Total ACLs performed amongst surgeons totalled 15,475.

The graft preference distribution can be seen in Table 1. No allograft or synthetic graft was used by any of the correspondents. When respondents indicated their preferred graft as "other," this was due to an equal/dual preference. Whenever dual preference existed, it was for hamstrings and either the quadriceps tendon or patellar tendon bone-tendon-bone (BTB).

**Table 1:** Distribution of preferred graft types among respondents (n=28) BTB: bone-tendon-bone

Graft Type	Hamstrings	Patellar Tendon BTB	Quadriceps	Other
Frequency/n (%)	18 (64)	4 (14)	2 (7)	4 (14)

Three orthopaedic surgeons (n=3, 11%) reported previous experience of graft contamination once in their career. The median annual ACLRs amongst these surgeons was 30 (range: 20-75), and the mean practice duration was 14 years. No more than one contamination was reported by the respondents. Decontamination with chlorhexidine gluconate (CG) was the most common method reported (100%), with one respondent reporting additional soaking of the graft in saline and vancomycin.

**Figure 1:**
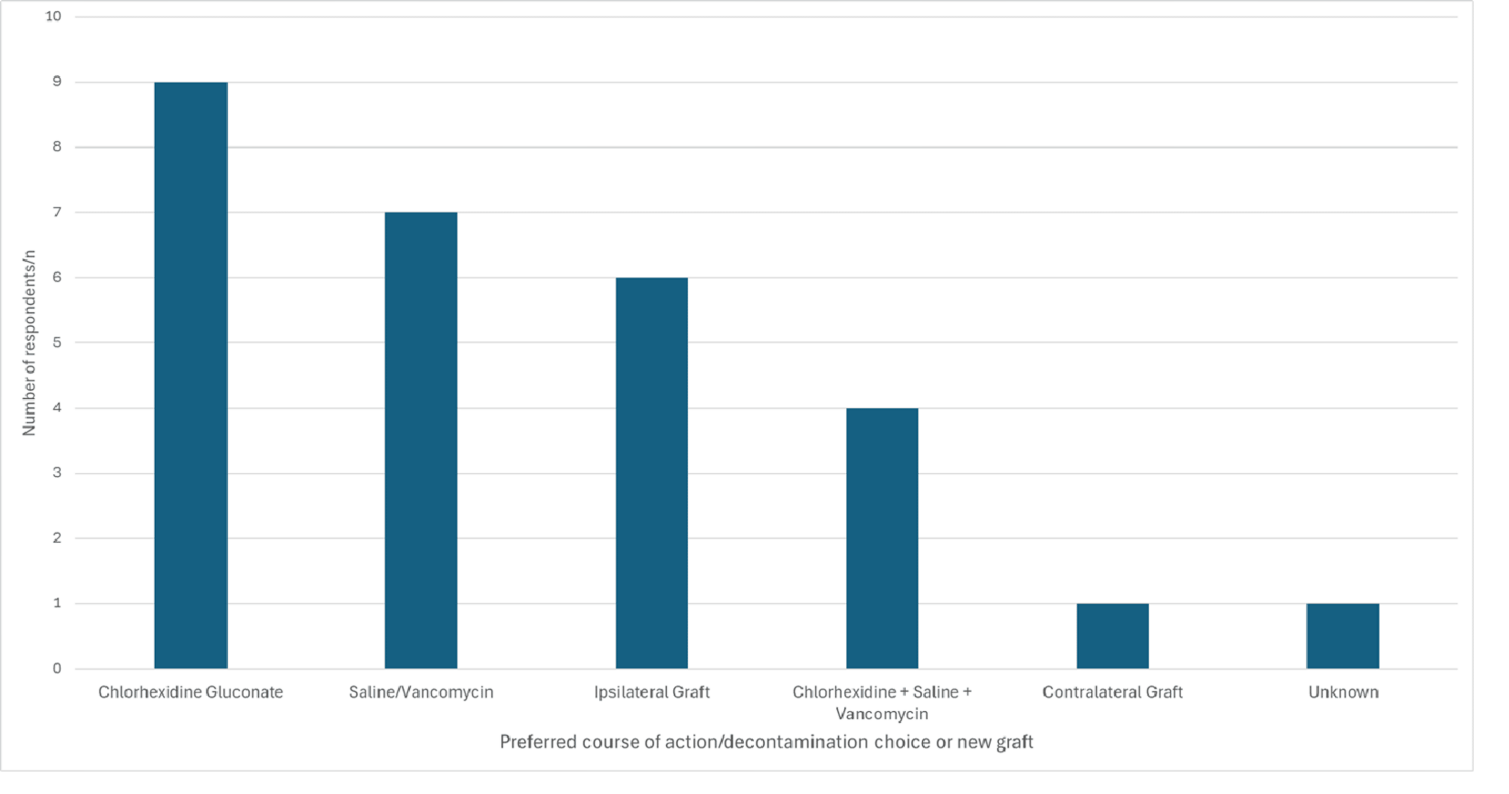
Bar chart demonstrating the preferred management strategies for intra-operative graft contamination

Responses to the hypothetical scenario of intra-operative graft contamination can be seen in Figure 1. Seven (33%) respondents would harvest another graft, whereas 21 (66%) would undertake a form of decontamination.

The awareness of various aspects of literature on the impact of intra-operative graft contamination and decontamination varied amongst the respondents and is summarised in Table 2. Of the 17 total respondents who indicated an awareness of literature in any category, 15 (88%) confirmed that this would influence their planned actions, or had influenced their actions when a graft was dropped in the past. 

**Table 2:** Proportions of respondents answering "Yes" to indicate knowledge of the literature in key areas of graft contamination

Topic	Mechanical Properties	Bacterial Load	Postoperative Infection	Clinical Outcomes
Frequency/n (%)	15 (54)	10 (36)	11 (39)	15 (54)

## Discussion

This cross-sectional study provides insight into the current strategies among UK orthopaedic surgeons for managing intra-operative ACL graft contamination. There are no currently established guidelines on how to manage intra-operative graft contamination. We report an 11% prevalence within our cohort of 28 surgeons. In the most recent report of the UK national ligament registry in 2022 [12], 136 surgeons contributed 1130 cases, and in the ACL surgery necessity in non-acute patients (SNNAP) study carried out in the same year, an estimated annual 30,000 ACLR cases were being performed in the UK [13]. There are 6,869 orthopaedic surgeons (including trainees) in the UK based on 2021 data [14] and 2,654 consultant-grade surgeons in the NHS in England currently [15]. A much smaller number of these professionals will be routinely performing ACLR, but if 11% encounter inadvertent graft contamination, this represents a significant number of cases.

Hamstrings as the primary graft choice were in keeping with modern global trends [16], and a third of respondents in our survey would harvest another graft rather than decontaminate. Other evidence reports only 18% of respondents would do so [3], although this paper was published 21 years ago, and in such time, there has been a shift from BTB grafts more towards hamstrings [17]. Specifics of graft preparation were beyond the scope of our survey, but it is common practice to add more suture material to hamstrings grafts to ensure uniform tension and shape across a multi-strand construct. This is generally not performed as commonly in BTB or quad grafts. Previous papers have recommended removal of all foreign material, including suture from grafts, prior to decontamination [18], and the majority of experimental/in vitro studies have been performed on pieces of tendon without suture [6-11]. The difficulty in removing all suture material from the graft, as well as potential concerns for biomechanical properties following decontamination and re-suturing, may be involved with this finding.

Despite this, most surgeons who previously experienced intra-operative graft contamination opted for decontamination with chlorhexidine gluconate solution, with one using additional soaking in saline and vancomycin and another using both. This suggests that decontamination of the original graft is an acceptable approach amongst UK surgeons. There have been mixed messages from in vitro and animal studies [19,20] concerning the effect of decontamination on biomechanical graft properties. However, following chlorhexidine treatment, human patellar tendon grafts appear not to undergo significant changes in graft elongation, ultimate tensile strength, or stiffness [21].

Systematic review and meta-analysis evidence tells us that intra-operative graft contamination also occurs in 12.3% of uncomplicated ACLR cases involving hamstrings or BTB graft harvest in which there is no adverse event, such as dropping of the graft or desterilisation [22]. Additionally, it has been shown that pre-soaking grafts in vancomycin significantly reduces the postoperative risk of septic arthritis, from 0.34% to 0.05% [23] despite the rate of graft contamination during normal harvest. A reasonable number of surgeons surveyed indicated a preference for a new graft, with the majority opting for an ipsilateral graft where possible and some for a contralateral graft. This approach can eliminate concerns regarding contamination; however, it can lead to increased risks of donor site morbidity, prolonged rehabilitation, and potentially reduce functional outcomes depending on the nature of the additional graft taken. To our knowledge, there is no published evidence comparing outcomes of primary ACLR where a second graft has been harvested. Given the rate of ACLR graft contamination routinely without adverse events, with minimal rates of post-op septic arthritis with just a vancomycin soak, we suggest that harvesting of a second graft after inadvertent contamination is not necessary, provided that decontamination protocols are established and followed. The variability in approaches reflects the lack of established guidelines, underscoring the need for consensus. What should happen post-operatively in a situation where graft contamination occurs is also under-reported. Some recommend one to two weeks of prophylactic antibiotics and extra scrutiny within the first six weeks [18]. Further elaboration is outside the scope of this study; however, given the reported effectiveness of the decontamination methods, further work would have merit to show whether there would be clinical benefit in spending additional resources on antibiotics and increased frequency of clinic follow-up appointments.

Limitations of this study include a small sample size, which may not accurately represent national practice in the UK or internationally. It is also subject to recall bias and self-reported behaviours, which may defer and not reflect intra-operative decision-making. Clinical data from this study are also limited; hence, the conclusions drawn are predominantly based on expert opinion. Within the respondents surveyed, only a small proportion have had direct clinical experience of graft contamination, and as such, the conclusions of our study are also based on the appraisal of current literature.

## Conclusions

Graft decontamination with CG is the preferred option for intra-operative graft contamination amongst surgeons surveyed, but various strategies were identified, including harvesting a new graft altogether, despite good evidence showing the effectiveness of CG. This variation in opinion highlights the need for guidance in the management of intra-operative ACL graft contamination. Given the exploratory nature of this work, future prospective comparative outcome studies would be indicated to aid guideline delivery.

Further work is required to explore surgeon experience, decontamination strategies, and their implications for morbidity and patients' functional outcomes. Additionally, 88% of surgeons surveyed who were aware of supporting evidence report that the literature on this topic is important in their decision-making process, but with no unifying guidance, responses are understandably variable. International consensus would also help drive towards a plan of action for this uncommon situation, and a joint statement from national associations would serve as useful support for surgeons faced with these challenging circumstances.
